# Unveiling the Influence of AI Predictive Analytics on Patient Outcomes: A Comprehensive Narrative Review

**DOI:** 10.7759/cureus.59954

**Published:** 2024-05-09

**Authors:** Diny Dixon, Hina Sattar, Natalia Moros, Srija Reddy Kesireddy, Huma Ahsan, Mohit Lakkimsetti, Madiha Fatima, Dhruvi Doshi, Kanwarpreet Sadhu, Muhammad Junaid Hassan

**Affiliations:** 1 Medicine, Jubilee Mission Medical College and Research Institute, Thrissur, IND; 2 Medicine, Dow University of Health Sciences, Karachi, PAK; 3 Medicine, Pontifical Javeriana University Medical School, Bogotá, COL; 4 Medicine, Sri Venkata Sai Medical College and Hospital, Mahabubnagar, IND; 5 Medicine, Jinnah Postgraduate Medical Centre, Karachi, PAK; 6 Medicine, Mamata Medical College, Khammam, IND; 7 Medicine, Fatima Jinnah Medical University, Lahore, PAK; 8 Medicine, Gujarat Cancer Society Medical College, Hospital & Research Centre, Ahmedabad, IND; 9 Medicine, All India Institute of Medical Sciences, Bathinda, IND; 10 Internal Medicine, Faisalabad Medical University, Faisalabad, PAK

**Keywords:** deep learning, health care, machine learning (ml), predictive analytics, artificial intelligence

## Abstract

This comprehensive literature review explores the transformative impact of artificial intelligence (AI) predictive analytics on healthcare, particularly in improving patient outcomes regarding disease progression, treatment response, and recovery rates. AI, encompassing capabilities such as learning, problem-solving, and decision-making, is leveraged to predict disease progression, optimize treatment plans, and enhance recovery rates through the analysis of vast datasets, including electronic health records (EHRs), imaging, and genetic data. The utilization of machine learning (ML) and deep learning (DL) techniques in predictive analytics enables personalized medicine by facilitating the early detection of conditions, precision in drug discovery, and the tailoring of treatment to individual patient profiles. Ethical considerations, including data privacy, bias, and accountability, emerge as vital in the responsible implementation of AI in healthcare. The findings underscore the potential of AI predictive analytics in revolutionizing clinical decision-making and healthcare delivery, emphasizing the necessity of ethical guidelines and continuous model validation to ensure its safe and effective use in augmenting human judgment in medical practice.

## Introduction and background

The exponential expansion of healthcare expenses has surpassed the pace of gross domestic product (GDP) growth, creating a financially unstable situation for health systems worldwide. There was no uncertainty or confusion on this topic before the outbreak of the 2019 coronavirus illness (COVID-19) and the crisis in Ukraine. Several causes are fueling this problem, including scarce resources, a growing elderly population, an increase in chronic diseases, and the strain on healthcare facilities that were already overburdened by the high demand for their services. In addition, the health systems of other countries, such as Indonesia, Brazil, and India, are deteriorating because of the COVID-19 pandemic [1].

Health systems depend on evidence-based care strategies and robust disease management pathways to address demands and regulate behaviors in industrial healthcare delivery services. The term "HRO" refers to a highly reliable organization, which is characterized by the management of its services via either an "accountable care organization (ACO)" or a "health maintenance organization (HMO)." However, there has been an increase in the number of long-term health disorders in the United States. Approximately 60% of persons have to manage at least one chronic disease, while 40% have to cope with two or more. As a consequence, the country spends $3.3 trillion annually on healthcare expenses. Moreover, this situation underwent a significant transformation as a result of the emergence of a new viral disease, formally designated as COVID-19 by the World Health Organization on February 11, 2020, following its first detection in Wuhan, China, in 2019. Since then, there has been a significant transformation in healthcare due to the digital revolution, which will have a profound impact on several fundamental aspects of medical treatment [2]. This may be attributed to the immense burden that COVID-19 has placed on global healthcare systems, including its foundational infrastructure, supply chain, and workforce. The epidemic has forced healthcare stakeholders to use digital technologies. The healthcare sector saw substantial structural changes after the outbreak. For instance, the increasing popularity of virtual healthcare systems and associated digital technology has motivated present-day customers, or patients, to actively participate in healthcare-related decision-making [3]. However, formidable challenges may emerge; developing strategies to overcome them will clear the path for the advancement into the future of healthcare. Patients and their distinct experiences and expectations are the primary drivers of healthcare advancements. One of their main objectives is to spread patient-centered facilities worldwide via the advancement of technologically facilitated interactions between physicians and patients while customers may be hesitant to provide personal information [4].

In healthcare, the ability to accurately predict patient outcomes is crucial for providing timely and effective interventions. Traditional methods of risk assessment often fall short of capturing the complexity and dynamic nature of patient conditions. However, the emergence of artificial Intelligence (AI) predictive analytics presents a promising opportunity to enhance prognostic accuracy and improve patient outcomes. Despite the growing interest in AI applications in healthcare, there remains a need to comprehensively understand its impact on patient outcomes and identify areas for further improvement [5].

AI predictive analytics leverages advanced algorithms and machine learning (ML) techniques to analyze vast amounts of patient data, ranging from demographics and medical history to diagnostic tests and treatment outcomes. By identifying patterns and correlations within these data, AI algorithms can generate predictive models capable of forecasting patient outcomes with greater precision than traditional methods. Moreover, AI systems can continuously learn and adapt from new data, enabling them to evolve and improve over time [6].

While numerous studies have explored the potential of AI predictive analytics in healthcare, there is a noticeable gap in the literature regarding its direct impact on patient outcomes. Existing research often focuses on technical aspects such as algorithm development and performance evaluation, overlooking the real-world implications for clinical practice and patient care. Moreover, few studies have systematically evaluated the effectiveness of AI predictive models in improving specific patient outcomes across different medical conditions and care settings. This gap in knowledge limits our understanding of the full potential of AI in healthcare delivery and hinders the translation of research findings into actionable insights for clinicians and policymakers [7].

Predictive tools are automated and employ a wide array of sophisticated statistical techniques to predict the future occurrence of events from historical and current data. Such technologies are the key components in industries such as industry, health, and education due to their ability to improve the decision-making processes [8]. Predictive analytics makes use of learning algorithms, various statistical modelling techniques, and data mining technologies in order to draw inferences from the data and predict trends and behaviors based on the data [9]. The utilization of these technologies has been groundbreaking, resulting in the processes of digital transformation that have had sweeping impacts across software testing, educational management, and business operations [10]. In the medical area, predictive analytics can change the face of patient care by forecasting infectious disease outbreaks, tailoring treatment plans, and employing hospital resources with more effectiveness. Considering the fact that machine learning algorithms have the ability to forecast patient risks for particular ailments, early diagnosis and preventive care are made simple [11].

Various types of predictive analytics platforms are used in healthcare. These include not only health maintenance organizations but also new integrated networks of care providers designed on patient-centered healthcare approach. An example is the mentioned platform for the University of California (UC) San Diego Health System, which implemented a predictive analytics algorithm right into regular healthcare workflow. They take and analyze electronic health record (EHR) data and use deep learning (DL) models for the early detection of cases such as sepsis [12].

The PARAllel predictive MOdeling (PARAMO), a healthcare analytics platform, which uses EHRs to prompt and optimize the entirety of the predictive modelling throughout patient cohorts, is playing a primary role in the face of a healthcare crisis. Thus, this speedy software has heavily sped up the computational time of modelling tasks by parallel processing the jobs [13].

Different platforms choose artificial intelligence implementations to study large volumes of patient records to predict future diseases. These platforms deeply confront vulnerability goals such as data privacy and algorithmic bias, but they also improve personalized care and the monitoring and management of diseases at the same [14].

This comprehensive narrative review aims to bridge the gap between AI technology and patient outcomes in healthcare. By synthesizing existing literature and empirical evidence, this paper will provide a thorough examination of the influence of AI predictive analytics on various aspects of patient care, including diagnosis, treatment selection, risk stratification, and prognosis. Furthermore, by critically analyzing the strengths and limitations of current AI applications, this review will identify key challenges and opportunities for future research and implementation. Ultimately, this paper seeks to offer valuable insights to healthcare professionals, researchers, and policymakers seeking to harness the power of AI to improve patient outcomes and advance the quality of care delivery.

## Review

Response prediction by AI

**Figure 1:**
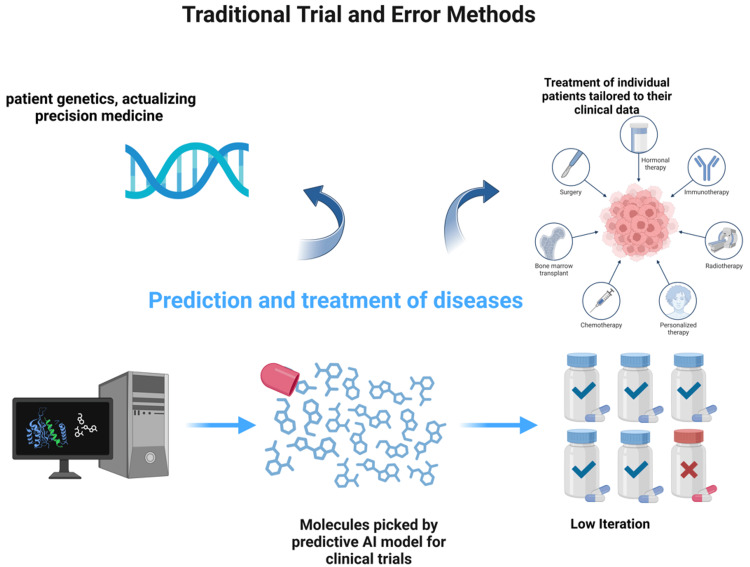
Outcomes of selected and predicted treatment using AI Created with biorender.com and extracted under premium membership AI: artificial intelligence

Artificial intelligence has the potential to assist in evaluating how patients react to medical treatment. By extracting data from electronic health records, intelligent algorithms can be developed to predict the outcomes of different treatments. This technology could help anticipate responses to therapy, determine appropriate drug dosages, and assess a patient's prognosis. Ultimately, it enables the identification of personalized treatment plans for each individual. These techniques are based on machine learning, a branch of artificial intelligence that involves recognizing and analyzing patterns in complex datasets [4]. Deep learning, a subset of machine learning, automatically classifies data without the need for explicit programming. These learning models may be used in everyday clinical practice to direct the development of therapeutic treatments and predict results. When implemented correctly, these procedures may significantly improve accuracy and decrease the time and expense associated with medication development and patient response evaluation (Figure 1) [5,6].

Artificial intelligence techniques in disease diagnosis and prediction

The field of artificial intelligence includes several branches of mathematics and science. The term "artificial intelligence" may refer to a wide range of computer-generated tasks that are given the impression of "intelligence" [7]. In order to train AI systems, data representing populations are used [15,16].

**Figure 2:**
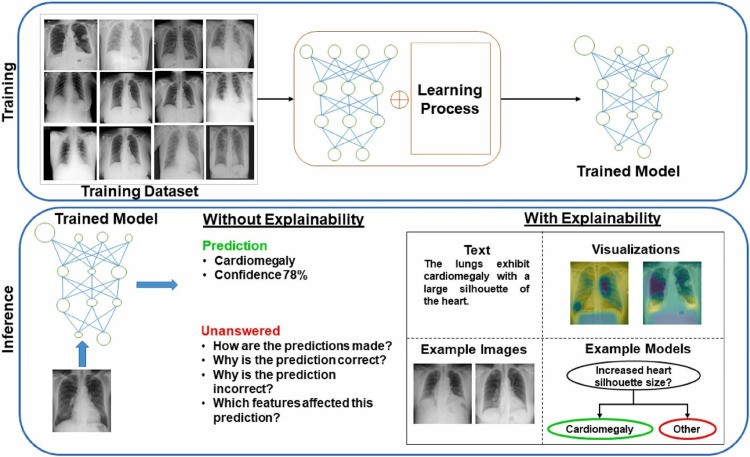
XAI helps stakeholders to understand the model's decision Reproduced under the terms of the Creative Commons CC-BY license from Nazir et al. [19]. Copyright © 2023 The Authors, published by Elsevier Ltd. XAI: explainable AI

A computer can learn on its own with the help of input datasets, experience, and feedback information; this is the goal of machine learning [17]. The machine learning algorithm learns to maximize its accuracy in a given task by analyzing the feedback it gets. The end goal is for it to work correctly on both new and old datasets [18]. One of the most common tools used to gather diagnostic data about patients is an image source. Nevertheless, this method is vulnerable to increasing resource restrictions and relies on human interpretation. One effective way to deal with the problems caused by human error due to ignorance or the lack of training is to apply artificial intelligence, specifically deep learning, in the field of medical imaging. Artificial intelligence plays a crucial role in image-based illness classification, computer-aided design (CAD), and disease segmentation. Diagnosing medical imaging procedures requires a training-based approach since basic equations cannot adequately reproduce images of tissues and organs inside the healthcare system. Explainable AI (XAI) techniques aim to provide additional information about a model's decision, thereby improving trust in the model's decisions, as shown in (Figure 2) [19].

Machine learning for image-based illness diagnosis

**Figure 3:**
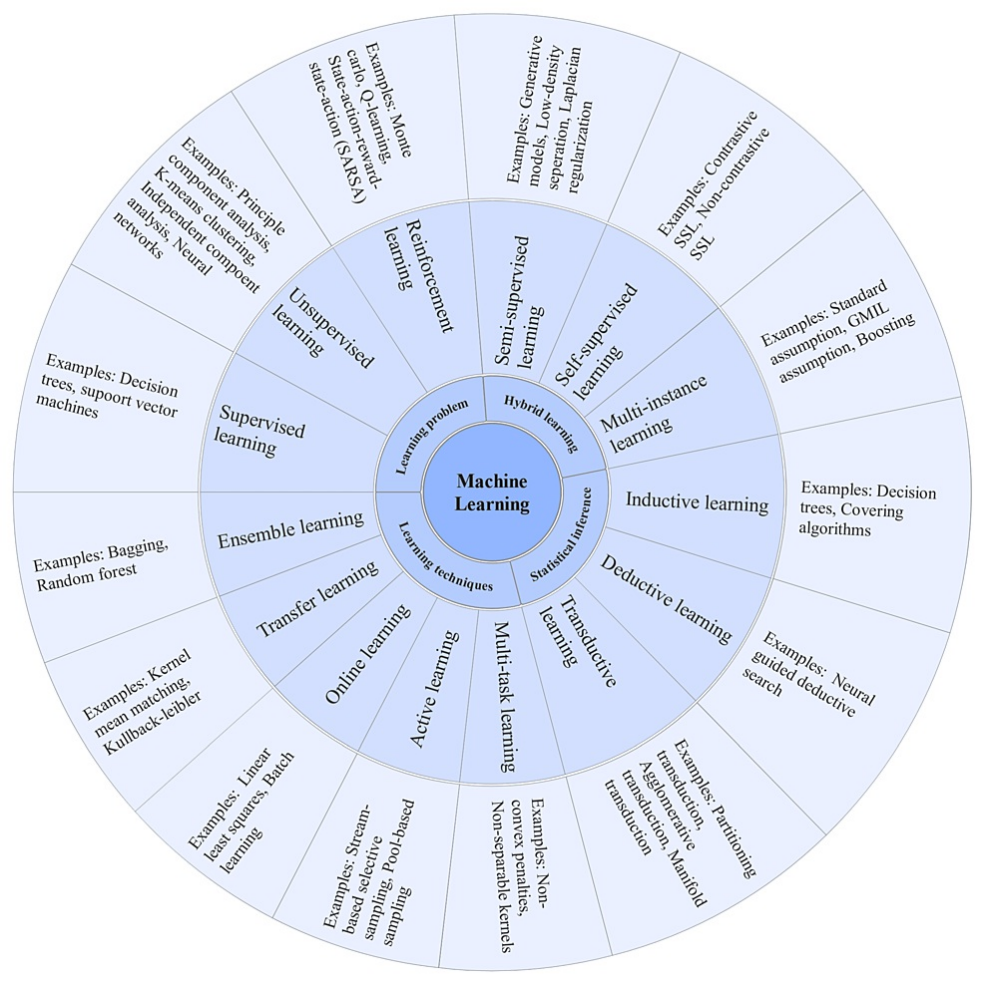
Different types of machine learning algorithms Reproduced under the terms of the Creative Commons CC-BY license from Ahsan et al. [24]. Copyright © 2022 The Authors, licensee Multidisciplinary Digital Publishing Institute (MDPI), Basel, Switzerland SSL, self-supervised learning; GMIL, Google multi-instance learning

The versatility of machine learning algorithms makes them useful in many contexts [20-22]. The field of medical imaging analysis is one that is seeing fast growth and great promise in the study of ML, a subfield of AI. There are many applications of machine learning in computer vision, CAD, and image processing for disease diagnosis [23]. Medical imaging has come a long way thanks to the merging of several imaging modalities, such as multiple-incision computed tomography (CT), positron emission tomography, tomosynthesis, magnetic resonance imaging (MRI), tomography, and diffuse optical tomography. This has led to an increase in the need for cutting-edge ML techniques for medical imaging analysis (Figure 3).

Machine learning is an umbrella term for a group of algorithms that can automatically spot patterns in data and use those patterns to predict future data or make decisions in uncertain environments. A distinguishing feature of machine learning is its dependence on data-driven methods, with minimum intervention from humans in the decision-making process. Input new data causes the software to learn from its training data and provide predictions [25].

A number of recent machine learning methods have been applied to the problem of disease prediction and detection [26,27]. In order to extract relevant data that may be used for disease prediction or diagnosis [28], we provide explainable AI methods such as SHapley Additive exPlanations (SHAP) to help us analyze the biggest significance [29]. The creation of synthetic medical images has prompted the development of generative models such as generative adversarial networks. Current data, such as lung sickness, may be improved with these pictures, and the findings can be performed better [30]. These approaches work well with one another and may be used to boost the model's efficiency. The specifics of the problem and the data dictate the methodology to be used [31].

Deep learning for image-based illness detection

One of the most resilient approaches is deep learning, an advanced piece of technology capable of autonomously learning several characteristics and patterns. Advancements in deep learning have made it possible to create prediction models that can identify diseases at an early stage. Deep learning algorithms surpass traditional machine learning approaches owing to their vast data analysis capabilities, automatic feature extraction, and outstanding accuracy, which scientists use using well-established pattern analysis methodologies. There is clear evidence that DL algorithms outperform ML when it comes to handling massive datasets. In addition, deep learning is said to have better anticipated accuracy than humans, which makes it the best method for dealing with images [32]. The major focus of deep learning is to extract useful information from images for diagnostic reasons, which has led to considerable interest in the medical area in relation to image processing. One typical use of DL algorithms is medical image-based diagnosis. Some examples of algorithms in this category are recursive neural networks, deep belief networks (DBNs), deep automatic encoders, deep Boltzmann machines, and deep intense normal machine learning [33].

One deep learning tool used for medical data analysis is the RAGCN, which stands for region aggregation graph convolutional network. In order to efficiently merge and condense data from different parts of an image, it makes use of graph convolutional networks (GCN). The program is designed to analyze medical pictures, including CT and MRI scans, which often show several regions of interest (ROIs) that need separate analysis. RAGCN uses a graph-based approach to divide the image into separate areas, and then, GCNs are used to extract features and provide predictions for each area. A method for automatically determining bone age using convolutional neural network (CNN) and GCN was presented in the work by Li et al. [34]. In feature extraction, convolutional neural networks were utilized, and for the inference of bone critical locations, GCNs were used. Combining these two distinct network topologies allowed researchers to build a novel graph convolutional network capable of analyzing the characteristics of the bone age assessment area. A deep learning method created with the express goal of detecting and classifying anomalies in medical imaging is the lesion-attention pyramid network. To efficiently extract important features from photos of varying sizes, the Local Appearance-based Parts Network (LAPNet) model uses a pyramid-based design. Additionally, it concentrates on areas of the picture most prone to lesion formation via an attention mechanism. In order to determine the extent of diabetic retinopathy, the writers in used this technique. In order to teach LAPNet to detect lesion regions, a large dataset of medical images was used (Table 1) [35].

**Table 1:** AI predictive analytics in patient outcomes AI: artificial intelligence

Factors	Identify high-risk patients	Early intervention and prevention	References
Disease risk	Predict disease progression	Tailored treatment plans	[36]
	Analyze genetic data	Personalized medicine approaches	[5]
Treatment	Predict drug response	More effective treatments and fewer side effects	[37]
	Real-time patient monitoring	Early detection of issues and treatment adjustments	[38]
	Readmission risk prediction	Reduce hospital readmissions	[39]

Artificial intelligence in recovery rate prediction and predicting complications

A study conducted among patients with acute kidney injury (AKI) in the ICU, for predicting the recovery and reversibility of renal function, showed that a machine learning algorithm used in the study had a higher quality in predicting the prognosis of AKI among the ICU patients in comparison with the models of traditional regression. It was concluded in the study that by using machine learning, we can potentially improve the prognoses of those critically ill patients by helping and assisting clinicians in providing timely interventions [40]. In a study about the use of machine learning (ML) in the pediatric population for estimating the aftereffects of traumatic brain injury (TBI), which is a major cause of fatality and disablement particularly in children, the conclusion was that machine learning algorithm is highly sensitive and can be used for counselling the prognosis of pediatric TBIs and can be of great significance as a screening tool in predicting useful results [41].

Artificial intelligence (AI) was also found to be very useful in evaluating postoperative recovery, which is a potential element in perioperative care, because it helped in determining an appropriate discharge date and in detecting complications [42]. As no universal definition exists, postoperative recovery is very challenging to evaluate and hard to predict. The research paper conducted among perioperative care oncology patients, assessing data from wearables about machine learning for the purpose of postoperative continuous recovery score, showed that ML can be an excellent tool for decision support [43].

Another study in patients undergoing major abdominal surgery for predicting surgical complications using AI concluded that the AI algorithms were very useful [42]. Diabetes is a condition characterized by the dysfunction of glucose homeostasis and is one of the most deadly and widespread chronic diseases of the modern era. A study done on diabetes with the help of artificial intelligence-powered tools indicated that AI techniques are being widely accepted as appropriate for the self-management of diabetes and thus can be utilized in daily clinical practice. The patient's quality of life was improved by them [44]. Although AI and ML techniques in cardiovascular medicine require further refinement and evaluation, there is a potential role of AI in providing clinical risk prediction, automated imaging interpretation and automated data extraction, and quality control. For predicting the risk of aortic aneurysm, coarctation, dissection, and atherosclerotic disease on computed tomography and MRI, multiple ML techniques have shown potential [45]. The deep learning (DL) algorithm with real-world data (RWD) was used in another study for predicting the practicability and implementation of total hip replacement (THR). It was shown that for assessing hip degeneration and speculating the requirement for further THR, the DL algorithm can bring forth a precise and dependable method. Also, RWD validated the role of DL in saving time and cost and offered alternative support for the algorithm [46]. Postexercise heart rate recovery (HRR) is a significant marker in assessing cardiac autonomy function. Any abnormality of HRR could be linked with adverse effects. A study with deep learning-derived estimates of HRR using resting electrocardiogram tracings was done to recognize individuals with threatened HRR [47].

Current immunotherapy outcome prediction methods

AI Predicts Immunotherapy Effectiveness From Histopathology

The gold standard for tumor detection is histopathological tissue sections. Their wealth of useful information may be used to track the course of the disease, choose individualized treatment plans, and predict the patient's chance of survival. However, traditional histopathology procedures are not up to snuff when it comes to precision medicine because of how much work experts need to put in to extract data from complex images [47]. Currently, digital pathology powered by AI has shown to be useful in the field of tumor diagnosis and treatment [48]. As an example, AI can accurately measure the results of immunohistochemical labeling and is able to separate and recognize cancer cells on histology slides. Hence, novel ways to predict tumor immunotherapy efficacy may be found by the use of machine learning techniques grounded on histopathology analysis [49]. Microsatellite instability, tumor-infiltrating lymphocytes (TIL), tumor-stroma ratio, and immunohistochemistry analysis are a few of the extensively researched topics.

By adhering to programmed cell death protein 1 (PD-1) on T cells, tumor cells expressing programmed death-ligand 1 (PD-L1) may suppress the immune response. As a method for combating tumors, immune checkpoint inhibitors (ICIs) work by blocking this contact. Immunotherapy efficacy and clinical outcomes are positively correlated with PD-L1 expression levels, according to the research. Previous research has shown that an AI-powered analyzer based on PD-L1 tumor ratio score may predict the success of immunotherapy better than a pathologist can in diagnosing non-small cell lung cancer. The results of this analyzer are also objective and reproducible; thus, they are free from the influence of human error. In addition, somatic genomic mutations accumulate due to a defective DNA mismatch repair (MMR) process that is spread in the *MMR* gene by mutations. The immunological checkpoint block response is highly associated with these mutations. For colorectal malignancies with MMR proficiency, the immune-related objective response rate was 0%, but for colorectal cancers with MMR deficiency, it was 40%. Based on these results, the MMR status may be able to predict how patients would react to immune checkpoint inhibitor therapy. Furthermore, several studies have shown that increased immune checkpoint blockade (ICB) is associated with higher T-cell infiltration and T-cell numbers [50].

One definition of a "microsatellite" is a small, repeated DNA sequence found within a genome. Microsatellite instability is a key factor in the development of several malignancies and is intricately related to DNA mismatch repair. In cancers with increased microsatellite instability, immunotherapy has shown promising results. Deep learning has potential for accurately and effectively determining if patients are immunotherapy candidates, according to research. To highlight the spatial organization of different cell types, it may subsequently use three-dimensional reconstruction. This is an extra factor that might be taken into account when determining how well immunotherapy works [51].

**Figure 4:**
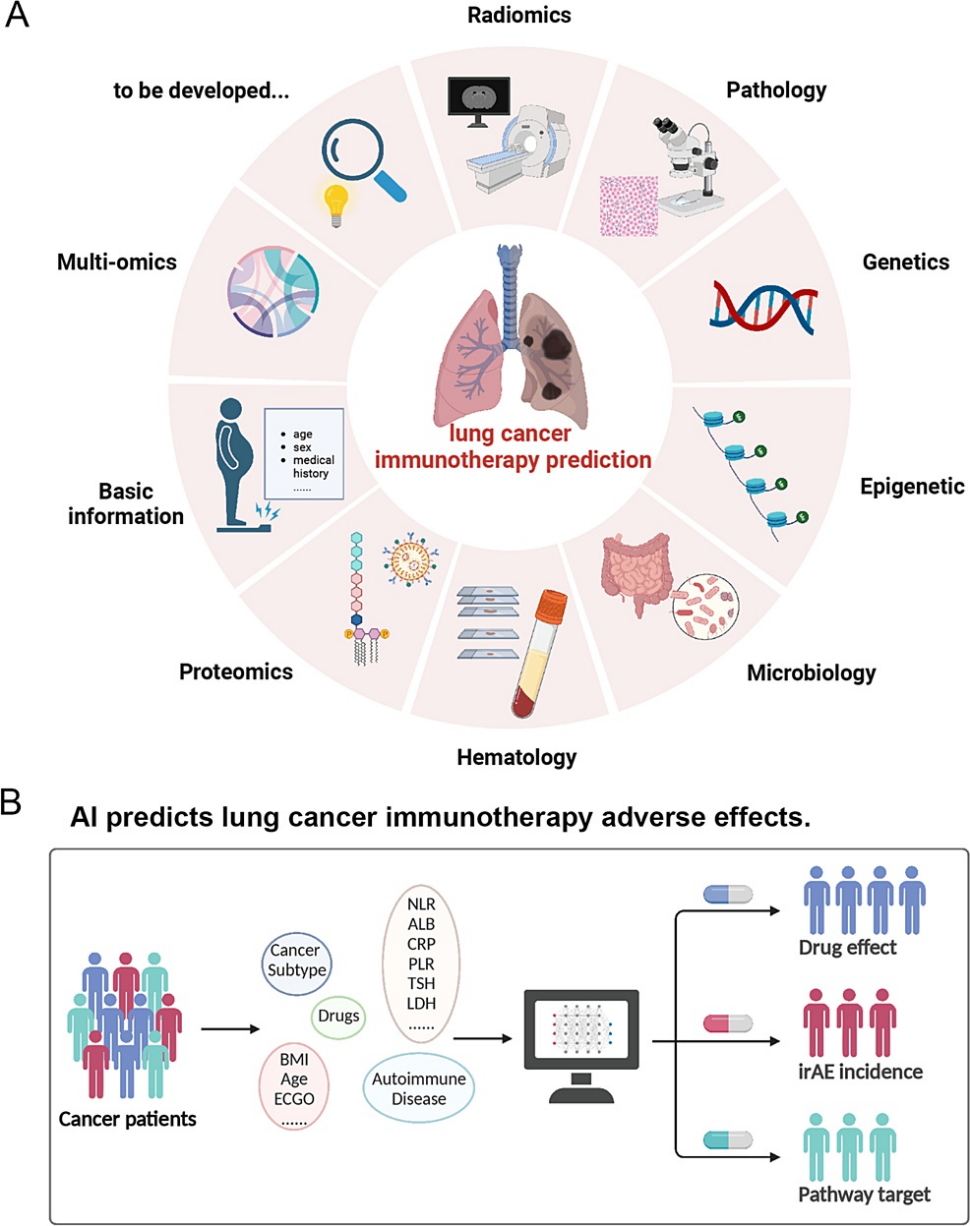
(A) Lung cancer immunotherapy prediction methods. The utilization of AI-based technologies in lung cancer immunotherapy prediction involves the analysis of various data types such as radiomics images, pathology images, genetic information, epigenetic information, microbiology data, hematology values, proteomics data, and multi-omics data. By leveraging diverse datasets, AI can effectively predict the benefits of immunotherapy in lung cancer patients. (B) The prediction of adverse effects on lung cancer immunotherapy Reproduced under the terms and conditions of the Creative Commons Attribution 4.0 International License from Gao et al. [54]. Copyright © 2023 The Author(s), published by Springer Nature AI, artificial intelligence; irAE, immune-related adverse event; NLR, neutrophil-to-lymphocyte ratio; ALB, albumin; CRP, C-reactive protein; PLR, platelet-to-lymphocyte ratio; TSH, thyroid-stimulating hormone; LDH, lactate dehydrogenase; ECGO, Eastern Cooperative Group for Oncology

There is a substantial correlation between immunotherapy and tumor-infiltrating lymphocytes, and models trained on hematoxylin and eosin (H&E)-stained images may reliably predict where TIL will be found. Researchers created an immune phenotype classifier that is related to prognosis [52]. Immunotherapy seemed to work better for phenotype A, which showed higher amounts of ICB. And there is a complete method for using response or outcome data to train deep learning models. To predict how the immune system would react to immunotherapy, this method employs graph neural networks or convolutional neural networks. The trials produced area under the curves (AUCs) for melanoma responder prediction at 0.69 and lung cancer responder prediction at 0.778 (Figure 4) [53].

Cancer cells' abundance of non-synonymous single-nucleotide variants (NsSNVs) is known as tumor mutational burden (TMB). The translation of these NsSNVs into unique antigenic peptides and the subsequent surface presentation of these peptides on cells may activate T-cells [55]. One predictive biomarker for lung cancer is elevated TMB levels. Recent research has shown that deep learning algorithms can predict how the immune system would react. Using H&E-stained photos, the study was able to predict TMB status, which was much better than the performance obtained by using solely clinical data [56].

With the use of pathology data, artificial intelligence has come a long way in predicting how tumor immunotherapy will work. Precision medicine and other medical breakthroughs may be possible as a result of the algorithm's standardization and the sharing of its results.

AI Predicts Immunotherapy Effectiveness Using Imaging-Omics

Medical pictures' capabilities have been greatly enhanced by the ongoing development of imaging equipment and technology, allowing them to go beyond the traditional "computer-assisted diagnostics format." These pictures now include data that can be mined with great throughput but are invisible to the naked eye. With the application of artificial intelligence, imaging histology is able to analyze and classify pictures with a degree of detail that surpasses that of individual graphics. This method enables the display of cellular, molecular, and macroscopic features [57]. In addition, prediction models powered by AI may one day provide trustworthy non-invasive markers for gauging immunotherapy efficacy. As a biomarker in immunotherapy, PD-L1 expression has been well-established. By integrating CT radiomics with clinical variables, a non-invasive evaluation of PD-L1 expression levels may be accomplished. From phase I clinical trials focusing on PD-1/PD-L1 monotherapy, 135 patients with malignancies localized at diverse locales were selected for the research [58]. Combining traditional CT scans with RNA sequencing (RNA-Seq) genetic data collected from tumor biopsy samples allowed for the development of a predictive imaging model [59]. In order to forecast the possible long-term therapeutic advantages of immunotherapy for these patients, the researchers developed multiparametric imaging histological signature models. To determine if multisource models may be more effective than radiomics-only models, more research is required.

There is consensus that TMB is a key indicator of ICI's efficacy. In order to find TMB radiomics indicators, researchers used deep learning techniques to CT images of patients with advanced non-small cell lung cancer [60]. Overall survival, progression-free survival, and responsiveness to immune checkpoint inhibitor treatment are all significantly predicted by these biomarkers. Researchers also used AI methods to look at pre-treatment-improved CT image analysis of PD-1-treated patients with non-small cell lung cancer and progressing malignant melanoma. Their research showed that immunotherapy was more likely to be effective against lesions with more morphological heterogeneity, defined as those with compact margins and inhomogeneous density.

A bad prognosis is linked with hyperprogression, when cancers develop fast after immunotherapy. However, there are currently few proven biomarkers to identify those at risk of this [61,62]. An investigation was carried out using clinical and imaging data from 109 patients with advanced non-small cell lung cancer who were given PD-1/PD-L1 immunosuppressant monotherapy [63]. The patients were all diagnosed with the disease. Hyperprogression was seen in 19 of these cases. The researcher retrieved the textural features from the patients' baseline CT images; these characteristics reveal the texture inside and around the target lesions. Furthermore, histological characteristics that quantify peri-lesion vascular tortuosity were retrieved. These characteristics may be able to help determine if a patient may have hyperprogression.

Most studies' radiomics quality scores were in the 11-20 range, with a high of 36 points [64]. The results raise the possibility that imaging-omics analysis powered by AI might one day shed light on the temporal and spatial variations present inside malignancies. This approach is very beneficial for predicting immunotherapy response, biomarker expression, and patient prognosis, especially in situations when histopathology materials are not accessible. The development of precision medicine, the assessment of disease risk, and the selection of immunotherapy-eligible individuals could all benefit from more research into imaging histology.

AI Predicts Genomic Immunotherapy Efficacy

Thanks to developments in sequencing technology, a mountain of cancer genetic data has been amassed, allowing for more precise recommendations to steer tumor treatment. The development of next-generation sequencing technology has allowed for the possibility of doing extensive genomic and transcriptome screening. Because of this, databases can be built to analyze tumor drivers, and cancer cells, stromal cells, and immune cells inside the tumor microenvironment can be sequenced to find out what therapy impacts are like. With an estimated three billion base pairs, the human genome is a massive dataset rich with complex information and many aspects. A comprehensive knowledge of the genome may be achieved by whole genome sequencing. However, deep learning approaches and artificial intelligence are required for gene identification and analysis, as well as phenotypic analysis and the inter-regulatory interactions between these variables [65]. Their approach successfully predicts both "hot" and "cold" immune system patients. Data from clinical trials were used to evaluate the model for external validation. Both general health and immunotherapy efficacy were higher in the hyperimmune group. A total of 110 individuals afflicted with metastatic melanoma were assessed by means of RNA sequencing and whole exome sequencing. Investigators found a link between cytolytic markers and the response rate to anti-cytotoxic T-lymphocyte-associated antigen-4 (CTLA-4) and TMB. In triple-negative breast cancer patients, there is a substantial correlation between levels of platelet-related genes, immunotherapy response, and prognosis [66]. The various responses to immune checkpoint inhibitors and the identification of prognostic markers may now be explored thanks to these studies.

Certain transcriptome components must also be included in genomic analysis for accurate immune response prediction and drug resistance understanding. A study found immunogenic mutant peptides with major histocompatibility complex (MHC) specificity by integrating exome and transcriptome sequencing with mass spectrometry. One possible use of these peptides is in the development of personalized vaccinations. To further explore tumor immunological interactions, Mo et al. developed a 384-well plate-based high-throughput screening technology [67]. This was accomplished by co-culturing cancer cells with peripheral blood mononuclear cells in each well. The goals were to assess cellular viability and to discover phenotypes related to cell proliferation. Furthermore, various bioactive compounds were tested for their effects, and three potential antagonists that might improve immune function were identified. The effectiveness of cellular immunotherapy in patients with B-cell malignancies is influenced by the DNA methylation patterns of cluster of differentiation 19 (CD19)-targeted chimeric antigen receptor T (CART19) cells, as shown by epigenetic profiling [68].

Extra resources

Numerous studies have explored the potential of artificial intelligence in tumor immunotherapy, with a range of applications [69]. The current liquid biopsy technology detects DNA from circulating tumor cells, making it a more practical and versatile way to diagnose and treat cancers [70]. To predict how well immune checkpoint inhibitors will work, immunotherapy researchers are developing liquid genetic indicators. Artificial intelligence can detect and evaluate molecular biological data in liquid samples automatically [71]. Furthermore, several biomarkers may be utilized to rule out pseudoprogressive or hyperprogressive illness following immunotherapy [72]. These include interleukins, plasma cytokines, and DNA from circulating tumor cells [73].

Along with genomes and imaging, proteomics has been the subject of much research as a potential biomarker for tumor immunotherapy effectiveness evaluation [74]. Patients with metastatic melanoma may now benefit from an AI-powered blood proteomics test model that can anticipate their reaction to immune checkpoint inhibitors [75]. There is a lot of promise in using AI models grounded on multi-omics to forecast tumor treatment responses. By integrating data from several sources, such as genomes, transcriptomics, epigenomics, proteomics, and radiomics, multi-omics may construct a more complete picture of a disease. To better anticipate the next 90 days for patients with non-small cell lung cancer, researchers use deep learning [76]. In order to distinguish between patients who responded to immunotherapy and those who did not, the AI model was utilized [77,78].

In addition, tumorlike organs may generate immune-tumor interactions in vitro and closely mimic the tumor microenvironment, making them a suitable screening model for immunotherapy. To solve the problems with safety and personalization that come with conventional prediction approaches, artificial intelligence is being integrated into organoids. The goal of this integration is to provide a streamlined platform for many applications, such as tumor in vitro culture, growth analysis, drug screening, and tissue collecting [79]. By analyzing cell necroptosis index, as well as antigen presentation pathways, AI may be able to forecast immunological checkpoint block reactions [80,81]. The next section will discuss the use of prediction analytics tools in surgery applications.

Surgery applications

Hepatobiliary and Colorectal Surgery

Researchers created a model to predict the occurrence of complications in patients who had undergone colorectal, hepatic, and pancreatic surgeries using data from the National Surgical Quality Improvement Program (NSQIP). A total of 15,657 patients were included in the training dataset. Values of 0.76 for surgical site infection prediction and 0.98 for stroke prediction were attained using the model's area under the curve. When compared to the American Society of Anesthesiologists (ASA) and American College of Surgeons Surgical Risk Calculator (ACS-SRC), the ML models performed better, according to the researchers. In order to anticipate difficulties in patients undergoing pelvic exoneration for locally advanced or recurring colorectal cancer, prior research has used deep learning algorithms. A dataset with 1,147 patients was used to train an artificial neural network model. The AUCs that were calculated ranged from 0.61 to 0.79. The researchers found that their deep learning model outperformed logistic regression in predicting a result utilizing a complicated mix of patient and procedure-related variables [82].

Cardiothoracic Surgery

In order to predict the likelihood of major complications after coronary artery bypass graft and/or valve surgery, researchers used machine learning algorithms. With an area under the curve of 0.72, the random forest model outperformed the other models while testing data from 3,700 patients. To predict cardiopulmonary complications in patients following lung resection, a machine learning method was developed. With an area under the curve of 0.75 and an accuracy rate of 70%, the extreme gradient boosting model outperformed its predecessors using a dataset of 1,360 patients. Based on their investigation of patient-specific data, the researchers concluded that machine learning algorithms provide individualized predictions. Personalized postoperative care recommendations, preoperative regimen optimization for high-risk patients, and care quality assessment are other ways these models help with surgical decision-making [83].

Plastic and Reconstructive Surgery

Problems with implant-based breast reconstruction have been anticipated with the use of machine learning techniques. Predictions about periprosthetic infection and the need for device explantation were made using machine learning models trained on perioperative data collected from 481 patients. In terms of predicting infection (AUC, 0.73; accuracy, 83%) and the need for device explantation (AUC, 0.78; accuracy, 84%), our results show that the machine learning models performed quite well. Furthermore, when it came to identifying pertinent risk factors including device implantation plane, acellular dermal matrix type, and adjuvant therapy, machine learning models outperformed traditional multivariable logistic regression. Machine learning found nine infection predictors when given the same data as multivariable logistic regression, which only found two. By using these algorithms, surgeons may be able to make more educated decisions and provide patients with more accurate and unbiased information about their reconstructive alternatives and the risks and benefits of each. By projecting the benefits and drawbacks of a procedure, patients may be better able to get informed consent via the use of models. To further improve the patient's suitability for the treatment, these models may also reveal variables that can be changed prior to reconstruction [82,83].

Neurological Surgery

In order to foretell complications in patients undergoing brain tumor surgery, researchers developed the extreme gradient boosting model. The model demonstrated a 70% accuracy rate and an AUC value of 0.74 using a dataset consisting of 668 cases. In particular, its prediction power was higher than that of a conventional statistical model. In order to foretell complications after deep brain stimulation surgery, researchers performed further studies using machine learning approaches. The supervised models performed very well in differentiating between a number of issues, such as infection (AUC: 0.97), complications within 12 months (AUC: 0.91), the need for a second surgical procedure (AUC: 0.88), and any problem (AUC: 0.86). Neurosurgery patients may benefit from better risk assessment, preoperative informed consent, and treatment planning with the use of machine learning, according to the study's authors [84].

General Surgery

Machine learning approaches that can predict the efficacy of abdominal wall repair have recently been developed by our team. Seven hundred twenty-five patients' data were used to generate an ensemble of nine supervised ML models. To improve the accuracy of the predictions, our ensemble made use of a multitude of ML techniques. Over the course of 30 days, the ensemble using the majority rule forecasted hernia recurrence, surgical site occurrences (SSO), and readmissions. The machine learning models showed excellent predictive accuracy over a lengthy three-year follow-up period in predicting issues such as hernia recurrence (accuracy, 85%; AUC, 0.71). Others excelled on 30-day readmission (accuracy, 84%; AUC, 0.73) and SSO prediction (accuracy, 72%; AUC, 0.75). Model analysis also allowed us to see factors linked to negative outcomes that were hidden by more conventional statistical methods such as logistic regression. This study looked at a variety of factors, including the amount of wound contamination, the frequency of previous abdominal operations, and the types of surgical procedures. Using the same datasets, ML analysis found 12 predictors for single sign-ons, whereas multivariate logistic regression analyses found five drivers. In order to enhance surgical planning, preoperative optimization, and collaborative decision-making, ML models may provide valuable information [81-84].

Risk calculators

Medical professionals may provide real-time risk assessments using machine learning to determine whether a patient is in an ideal condition for surgical intervention. For the purpose of anticipating serious problems after surgery, two ML-driven risk calculators have just become accessible. The 51,457 patients who had undergone significant inpatient surgery were used to create and verify the MySurgeryRisk model. The eight primary postoperative complications that were expected to be predicted were acute renal damage, sepsis, venous thromboembolism, wound complications, neurological and cardiovascular difficulties, mechanical ventilation after 48 hours, and admission to intensive care after 48 hours. A further goal of the model was to predict mortality rates during the first two years after surgery. In terms of area under the curve, the model produced results between 0.77 and 0.94. Research evaluated the validity and reliability of the MySurgeryRisk calculator in comparison to physicians' clinical judgment. The study found that MySurgeryRisk was more accurate in predicting postoperative problems than doctors' initial risk assessments. Also, the risk assessments made by the doctors improved significantly when they started using the machine learning model [85]. An independent group of scholars created the POTTER risk calculator. A decision tree machine learning dataset of 382,960 NSQIP patients served as the basis for the model's training. The POTTER risk calculator outperformed the ASA, Emergency Surgery Score, and NSQIP in terms of predicting mortality and morbidity [86]. The next section will discuss the ethical considerations and challenges in the use of prediction analytics tools.

Ethical considerations and challenges

Ethical questions and data privacy problems constitute the main obstacles to AI implementation in healthcare, especially when you consider predictive analytics applications. Given that the use of AI in healthcare is rising, personal data protection, patient injustice, and accountability are among the noticeable worries. As AI inhabits patient data processing, data privacy is an imperative element when it comes to trust and to abide by regulations. Patient data security encompasses implementing robust measures for data protection against unauthorized access to patients' sensitive information, maintaining secure data storage, and putting into place data management processes that are clear and concise [87]. The health data of patients must be kept secure through the application of data protection regulation such as the Health Insurance Portability and Accountability Act (HIPAA) and General Data Protection Regulation (GDPR) to ensure that the system gets to stay within the rules and also the patients can trust it. Departmental implicit bias is yet another ethical issue when AI is applied. It involves the belief that a machine can substitute a doctor and make medical and ethical decisions. It can be that AI software, while unintended, creates biases, which, in turn, leads to inequality and, as a result, disparities in care and outcomes for some patient populations [88]. However, to succeed in this, AI innovation must give top priority to fairness and equity in algorithmic design so that AI systems are used for the benefit of all and do not widen the existing healthcare gaps. The fact that accountability is also a very crucial ethical concern in the implementation of artificial intelligence is something to be noted. Pinning the point of responsibility for AI-associated mistakes or downsides can be hard, considering that AI systems are likely to be combined with input by human expertise and decision-making systems [89]. The practical implementation of AI as a caregiver, therefore, requires that certain mechanisms are put in place to ascertain its safe use. Among others, clear lines of accountability and robust error detection and correction mechanisms are needed to attain this. Hence, by including these ethical concerns, providers of healthcare can better the quality of care and efficacy of healthcare delivery through AI-given predictive analytics while preserving patient autonomy and privacy [90].

## Conclusions

In the current era of technology, innovation is crucial, and the healthcare industry is keeping up with the trend. This paper examines several research that demonstrate the use of artificial intelligence (AI) and related technologies in predicting various aspects of a patient's trip. AI algorithms have a remarkable ability to serve as an excellent tool for enhancing personalized healthcare and boosting prognosis. Deep learning (DL) and machine learning (ML) have significantly transformed the field of detecting and forecasting disorders. Nevertheless, similar to every groundbreaking advancement, AI will inevitably bring about unintended repercussions and probable ramifications for the delivery of healthcare and existing hazards for patients. Over time, it is crucial to continuously improve the methods of collecting and validating data, as well as the ability to revise them as technology advances. Once implemented, constant monitoring is necessary to assess and modify the AI algorithm. This will also contribute to addressing ethical concerns. Artificial intelligence has the potential to greatly impact the healthcare industry by revolutionizing its practices, controlling costs effectively, and improving the experience of patients. Additionally, AI may assist physicians by providing them with data-driven insights and predictive analytics, enabling them to make more informed decisions.
